# Nerve Root Sedimentation Sign among Lumbar Canal Stenosis Patients Visiting the Department of Orthopaedics in a Tertiary Care Centre: A Descriptive Cross-sectional Study

**DOI:** 10.31729/jnma.7540

**Published:** 2022-12-31

**Authors:** Yugesh Raj Pant, Sushil Paudel, Rajesh Bahadhur Lakhey, Rohit Kumar Pokharel

**Affiliations:** 1 Department of Orthopaedics and Trauma Surgery, Tribhuvan University Teaching Hospital, Institute of Medicine, Maharajgunj, Kathmandu, Nepal

**Keywords:** *magnetic resonance imaging*, *prevalence*, *spinal stenosis*

## Abstract

**Introduction:**

Lumbar canal stenosis is a common cause of back pain and neurogenic claudication in the elderly population. Nerve root sedimentation sign-on Magnetic resonance imaging is a novel sign proposed for the diagnosis of lumbar canal stenosis. There is limited research so far. So, the aim of this study was to find out the prevalence of nerve root sedimentation signs in lumbar canal stenosis among patients visiting the Department of Orthopaedics in a tertiary care centre.

**Methods:**

This was a descriptive cross-sectional study conducted from 1 January 2020 to 31 July 2021 in the tertiary care centre, after receiving ethical approval from the Institutional ethical review board (Reference number: 299/(6-11)076/077). The anteroposterior diameter of the dural sac at the most stenotic level and nerve root sedimentation sign in magnetic resonance images were measured in patients with lumbar canal stenosis. Point estimate and 95% Confidence Interval were calculated.

**Results:**

Among 40 patients enrolled, 34 (85%) (84.01-85.99, 95% Confidence Interval) patients had positive nerve root sedimentation sign. Out of 34 (85%) cases with positive sedimentation signs, 32 (94.12%) had severe lumbar stenosis and the remaining 2 (5.88%) had moderate lumbar stenosis.

**Conclusions:**

The prevalence of nerve root sedimentation signs is similar to the similar studies done in similar settings. Nerve root sedimentation signs on magnetic resonance imaging can be used as an objective sign for the diagnosis of severe lumbar canal stenosis.

## INTRODUCTION

Lumbar canal stenosis (LCS) is a clinical syndrome of the buttock or lower extremity pain, which may occur with or without back pain.^1^ Radiological criterion on Magnetic Resonance Imaging (MRI) for the diagnosis of LCS is the anteroposterior diameter and cross-sectional area of the dural sac.^2,3^

The measurement of these parameters often correlates poorly with clinical findings in LCS.^4^ The nerve root sedimentation (NRS) sign is a radiological sign first reported by Barz in 2010.^5^ This newly proposed sign doesn't require measurement tools and can be easily used as an objective tool for diagnosing LCS in day-to-day practice.

Various studies have been done in the western population regarding the role of the nerve root sedimentation sign on MRI for diagnosis of lumbar canal stenosis with varying results,^4^ however, no such studies done in our setting are available.

The aim of this study was to find out the prevalence of nerve root sedimentation signs among lumbar canal stenosis patients visiting the Department of Orthopaedics in a tertiary care centre.

## METHODS

This descriptive cross-sectional study was conducted from 1 January 2020 to 31 July 2021 in a tertiary care centre in Nepal, after approval from the Institutional review committee (Reference number: 299/(6-11)076/077). Patients aged >40 years presenting to the Department of Orthopaedics with clinical-radiological features of lumbar canal stenosis (leg pain, back pain, or buttock pain, or neurogenic claudication or tingling or numbness in lower limb and MRI measurements of AP diameter <12mm in a dural sac at intervertebral disc level at and between L1-L2 to L4-L5 level and giving written consent were enrolled in the study.^2,3^

The patients with previous spine surgery, congenital spinal anomalies, peripheral arterial disease, or other musculoskeletal disorders with impaired walking ability, MRI-confirmed alternative diagnosis and with stenosis only at L5-S1 level were excluded from the study. Cases whose MRI scans showed stenosis at only L5-S1 level were also excluded as a protocol by Barz et al doesn't include L5-S1 level for evaluation of Nerve root sedimentation sign as the S1, S2 nerve roots exit ventrally from the spinal canal.^6^

The sample size was calculated by using the following formula:


$$\begin{array}{ll} \text{n} & ={\text{Z}}^{2}\times \frac{\text{p}\times \text{q}}{{\text{e}}^{\text{2}}}\\ & =1.96^{2}\times \frac{0.94\times 0.06}{0.08^{2}}\\ & =34 \end{array}$$

Where,

n = minimum required sample size
Z = 1.96 at 95% Confidence Interval (CI)
p = prevalence of patients with positive nerve root sedimentation sign taken from previous study, 94%^5^
q = 1-p
e = margin of error, 8%

The calculated sample size is 34. However, the final sample size taken was 40.

Anteroposterior (AP) diameter of the thecal sac at lumbar canal level ≤12 mm is considered radiological stenosis which is further divided into moderate (AP>10 mm and ≤12 mm) and severe stenosis (AP ≤10 mm).^2,3^ A positive sedimentation sign has been defined as the absence of nerve root sedimentation in at least one axial MRI scan, at a level above or below, disregarding the location of the scan within the level and its proximity to the maximal stenosis.^4^ The sedimentation sign is positive if there are nerve roots in the ventral part of the dural sac except for the ones exiting the dural sac. The sedimentation sign is measured at a level above or below the maximal stenosis because, at the level of the stenosis, nerve roots lie tightly packed in the dural sac and, therefore, cannot be identified and judged adequately.^6^

Proformas were filled which include the demographic data, history, VAS score, and the finding of sedimentation sign in MRI.

Data were entered in Microsoft Excel version 2007 and were analysed using IBM SPSS Statistics version 26.0. Point estimate and 95% CI were calculated.

## RESULTS

Among 40 patients enrolled, 34 (85%) (73.93-96.07, 95% CI) patients had positive nerve root sedimentation sign. The mean age is 58.64±11.17 years. A total of 22 (55%) were male and 18 (45%) were female (Table 1).

**Table 1:** Severity of lumbar canal stenosis among patients with positive nerve root sedimentation sign (n= 34).

Severity	n (%)
Moderate	2 (5.88)
Severe	32 (94.12)

All the patients 34 (100%) had low back pain and radicular pain. The mean duration of Low back pain was 7.48±7.04 years. The mean duration of radicular pain was 5.42±7.32 months, ranging from 1 week to 36 months. Total 2 (5%) out of fourty patients had loss of sensation over lower limb. None of the cases had bladder/bowel involvement. A total of 34 (80.95%) patients had low back pain which was aggravated by extension and relieved by flexion. The mean VAS score for pain was 4.88±1.47, ranging from 3 to 8.

Out of the 34 patients with positive nerve root sign, 12 (33.33%) had single level of spine affected and 22 (61.11%) had multiple level of spine affected. The most commonly affected level as defined by the minimum AP diameter of dural sac at axial MRI scan was L4-L5 which was affected in 80% of cases. The least commonly affected level was L1-L2 which was involved in 1 (2.78%).

## DISCUSSION

Lumbar canal stenosis is a common cause of back pain and neurogenic claudication in elderly population. Nerve root sedimentation sign is one of the novel signs described on MRI of lumbosacral spine for the diagnosis of LCS.^5,6^ In our study, nerve root sedimentation sign was positive in 85% patients with Lumbar canal stenosis. Nerve root sedimentation sign was positive in 94.12% of cases in severe stenosis group and 33.3% in moderate stenosis group. This is similar to the the findings of a study in which nerve root sedimentation sign was positive 80.8% of patient with severe stenosis and 13.8% patient with moderate stenosis.^7^ The overall positivity rate in this study is higher than that in the similar study which reported 56.8% overall positivity rate in LCS patients. This could be due to the enrollment of a smaller number of moderate stenosis cases as compared to the severe cases. The positivity rate in this study is lower than one of the study in which the positive nerve root sedimentation sign was seen in 97% of patient with LCS. The higher positivity rate of the NRS sign in the above study could be due to the enrollment of more severe cases of LCS in his study (Dural cross section diameter <80 mm^2^).^5^

Nerve root sedimentation sign is described only for supine MRI, so standing MRI cannot be analysed for NRS sign and NRS sign cannot be used to diagnose cases which have dynamic changes in lumbar canal anatomy causing LCS symptoms. Discrepancies between radiologic findings and clinical symptoms in LCS in this study could also originate from individual differences in sensitivity to back and/or leg pain.^8^

## CONCLUSIONS

Nerve root sedimentation sign on MRI in patients with severe lumbar canal stenosis in our study was similar to the other study done in a similar setting. Further study on the nerve root sedimentation sign and the morphological gradings on a larger population and multicentres is needed to establish the sensitivity and specificity of this novel sign which will help to rule in or rule out lumbar canal stenosis.

## References

[1] Watters WC, Baisden J, Gilbert TJ, Kreiner S, Resnick DK, Bono CM (2008). Degenerative lumbar spinal stenosis: an evidence-based clinical guideline for the diagnosis and treatment of degenerative lumbar spinal stenosis.. Spine J..

[2] Steurer J, Roner S, Gnannt R, Hodler J (2011). Quantitative radiologic criteria for the diagnosis of lumbar spinal stenosis: a systematic literature review.. BMC Musculoskelet Disord.

[3] Mamisch N, Brumann M, Hodler J, Held U, Brunner F, Steurer J (2012). Radiologic criteria for the diagnosis of spinal stenosis: results of a Delphi survey.. Radiology..

[4] Staub LP, Barz T, Melloh M, Lord SJ, Chatfield M, Bossuyt PM (2011). Clinical validation study to measure the performance of the nerve root sedimentation sign for the diagnosis of lumbar spinal stenosis.. Contemp Clin Trials..

[5] Barz T, Melloh M, Staub LP, Lord SJ, Lange J, Roder CP (2010). Nerve root sedimentation sign: evaluation of a new radiological sign in lumbar spinal stenosis.. Spine (Phila Pa 1976)..

[6] Schizas C, Theumann N, Burn A, Tansey R, Wardlaw D, Smith FW (2010). Qualitative grading of severity of lumbar spinal stenosis based on the morphology of the dural sac on magnetic resonance images.. Spine (Phila Pa 1976)..

[7] Zhang L, Chen R, Liu B, Zhang W, Zhu Y, Rong L (2017). The nerve root sedimentation sign for differential diagnosis of lumbar spinal stenosis: a retrospective, consecutive cohort study.. Eur Spine J..

[8] Lau YYO, Lee RKL, Griffith JF, Chan CLY, Law SW, Kwok KO (2017). Changes in dural sac caliber with standing MRI improve correlation with symptoms of lumbar spinal stenosis.. Eur Spine J..

